# Imaging Tests, Provocative Tests, Including Exercise Testing in Women with Suspected Coronary Artery Disease

**DOI:** 10.1007/s12170-012-0251-3

**Published:** 2012-07-15

**Authors:** Eleni Vavas, Susie N. Hong, Sonia Henry, Stacey E. Rosen, Jennifer H. Mieres

**Affiliations:** 1 Department of Cardiology, Hofstra North Shore-LIJ School of Medicine, 300 Community Drive, Manhasset, NY 11030 USA; 2 Division of Cardiology, Beth Israel Deaconess Medical Center, Boston, MA USA; 3 Department of Cardiology, Hofstra North Shore-LIJ School of Medicine, 270-05 76th Avenue, New Hyde Park, NY 11040 USA; 4 Hofstra North Shore-LIJ School of Medicine, North Shore-LIJ Health System, 1979 Marcus Avenue Suite 236, Lake Success, NY 11042 USA

**Keywords:** Noninvasive imaging, Sex-specific, Women

## Abstract

Evolving knowledge regarding sex differences in coronary heart disease has demonstrated that the prevalence, symptomatology, and pathophysiology of coronary atherosclerosis vary between genders. Women experience higher mortality rates and more adverse outcomes after acute myocardial infarction than men, despite a lower prevalence of obstructive coronary artery disease. Based on recent insights into the complex pathophysiology of coronary heart disease which includes a spectrum of obstructive coronary artery disease and dysfunction of the coronary microvasculature and endothelium, the term ischemic heart disease is a more accurate term for discussion of coronary atherosclerosis specific to women. In women, with clinical features and risk factors for ischemic heart disease, the detection and evaluation of ischemic heart disease is challenging due to the diverse pathogenic mechanisms of ischemic heart diseases in women. In this article, we discuss noninvasive imaging tests, provocative tests, including exercise testing in women with suspected ischemic heart disease.

## Introduction

Marked reductions in cardiovascular mortality in women have occurred for the first time in the past decade due to an increase in awareness, greater focus on prevention strategies as well as the application of evidence-based treatments for established coronary artery disease (CAD) [1–3]. Despite this progress, CAD is still the leading cause of death and disability in the United States, claiming the lives of almost 200,000 women every year [4–7]. The focus on sex-specific cardiovascular disease research has greatly improved our understanding of the pathophysiology of coronary disease in women [4]. The Women’s Ischemia Syndrome Evaluation (WISE) trial and related studies have expanded our understanding of the complex pathophysiology of coronary atherosclerosis in women to include abnormal coronary reactivity [8], microvascular dysfunction [9], and plaque erosion/distal microembolization [10, 11], as contributory factors to a female sex-specific ischemic heart disease [12•, 13, 14••]. Although prior evidence supports the fact that atherosclerotic plaque impeding forward flow in a coronary artery is the primary etiology of symptoms, morbidity, and mortality for coronary artery disease, data from the WISE trial elucidated a new paradigm for ischemic heart disease in women. In a cohort of the WISE women, chest pain and abnormal stress tests occurred without a critical (flow-limiting) lesion and at 5-year follow-up, these women had an intermediate risk of cardiovascular events. The WISE study provides prognostic information on symptomatic women with nonobstructive disease. In these women, symptoms persisted in the setting of usual anti-anginal therapy and during 5-year follow-up, there was an increased rate of cardiac mortality, myocardial infarction, stroke, and hospitalization for congestive heart failure for the 33 % of symptomatic women with angiographically normal or nonobstructive coronary stenoses compared with a similarly matched cohort of women without symptoms [15]. Endothelial dysfunction and microvascular disease was felt to be the cause of persistent symptoms and abnormal stress tests in this group of WISE women [14••].

Additional data reveal that younger and middle aged women with acute MI are more likely to have a poorer prognosis and higher mortality compared with their male counterparts [16–18], and that in over 50 % of symptomatic women undergoing cardiac catheterization for acute coronary syndrome (ACS), no evidence for obstructive CAD is found [14••]. Recent data have added to our understanding of the mechanism of ACS in women in the absence of focal areas of stenosis. Intravascular ultrasound and cardiac magnetic resonance (CMR) were used to demonstrate evidence of plaque disruption and myocardial damage among women with confirmed acute myocardial infarction (AMI) and normal or only minimally abnormal coronary angiograms [12•, 19]. In women, the dynamic interaction of hormonal fluctuations, risk factors, and differences in vascular structure and function contribute to the development of a somewhat different form of ischemic heart disease (IHD) disease with small coronary arteries, diffuse disease, and microvascular dysfunction being seen more frequently among women [4, 7, 14••]. Given the expanded milieu of coronary atherosclerosis in women, the term ischemic heart disease (IHD) is proposed as a more accurate and inclusive term for the spectrum of coronary atherosclerosis specific to women, rather than CAD or coronary heart disease (CHD) [14••].

These new data pave the way for an expanded role of cardiac imaging to investigate other elements implicated in the spectrum of IHD in women—the coronary and noncoronary atherosclerotic burden and methods to detect dysfunction of the coronary endothelium, flow reserve, and microvasculature—as these factors have increasingly significant implications for diagnosis, prognosis, and treatment [7, 14••].

Therefore, early identification of women at risk for ischemic heart disease is critical, especially given that sudden cardiac death is often the first manifestation of IHD in a high proportion of women (52 %), compared with men (42 %) [20].

We review the literature on the role of contemporary and emerging techniques for the diagnostic and prognostic evaluation of symptomatic women at risk for ischemic heart disease.

## The Role of Contemporary Testing Techniques for Diagnosis and Risk Assessment of Symptomatic Women at Risk for Ischemic Heart Disease

### Exercise Electrocardiogram (ECG)

Exercise stress testing is the most commonly used method of diagnosing IHD in women and is the initial noninvasive study of choice. As per the American College of Cardiology/American Heart Association guidelines, nonimaging treadmill exercise stress testing (ETT) continues to be an appropriate first line testing symptomatic women who are (1) deemed intermediate risk for IHD, (2) have a normal resting 12-lead ECG, and (3) are capable of maximal exercise [21–23]. Although historical evidence suggests a lower sensitivity and specificity of ETT for the detection of epicardial disease, recent research has improved our understanding of the diagnostic and prognostic value of this modality in women [24•].

Lower exercise tolerance of women and the use of traditional criteria for a positive ETT of ≥1 mm horizontal or downsloping reversible ST-segment depressions result in an underdiagnosis of obstructive coronary disease. In a meta-analysis of 3721 women being evaluated for IHD with an ETT, positive ECG changes were shown to have a sensitivity and specificity in women of 61 % and 70 %, respectively [25], compared with men where both sensitivity and specificity have been shown to be approximately 10 % higher [14••]. The accuracy for detecting obstructive CAD is dependent on the magnitude, morphology, and duration of the ECG changes. Marked ST-segment changes (ie, ≥2 mm horizontal or downsloping ST depression at low workloads and persisting into recovery) are more sensitive markers for epicardial disease in women [26].

The strength of the ETT lies in its ability to provide important prognostic data on the risk of cardiac death or myocardial infarction in women. The diagnostic and prognostic accuracy of exercise testing in women can be improved by incorporating parameters such as exercise capacity, chronotropic response, heart rate recovery, blood pressure response, and the Duke Treadmill Score (DTS), in addition to ST-segment depression with exercise [24•]. The DTS is defined as exercise time, (5 × ST segment deviation), 4 × chest pain (1 = nonlimiting, 2 = limiting) [27]. Duration of metabolic equivalents (METs) is the strongest prognostic variable from treadmill ETT [28, 29] with a higher death rate in women who can achieve less than 5 METS compared with those able to achieve more than 8 METs [30].

The powerful prognostic role of exercise capacity as determined by the ETT was validated in the recent publication of the prospective, randomized clinical trial “What is the Optimal Method for Ischemic Evaluation in Women (WOMEN) Trial.” In the cohort of 824 symptomatic women who were randomized to exercise treadmill test or exercise stress myocardial perfusion imaging, no difference in outcomes at 2 years was demonstrated in these symptomatic women with suspected epicardial disease and who were able to exercise [31••].

Therefore, exercise ECG is the test of choice for a symptomatic woman with a normal ECG who is capable of achieving 5 METS or more. The use of the Duke Activity Status Index (DASI) can help predict functional capacity. The DASI score, a weighted scoring system that uses a 12-question survey, has been validated in women to estimate the METs associated with the activities of daily living. When DASI was used in the WISE trial, there was an increased risk of cardiovascular death and nonfatal MI in women with a calculated DASI score of less than 5 METs [19].

### The Role of Stress Echocardiography

Contemporary stress echocardiography utilizing exercise or dobutamine can be used to identify stress-induced ischemia based on the development of regional wall motion abnormalities in the area of a decrease in myocardial blood flow. Its role in symptomatic women at risk for ischemic heart disease has been well established. Cumulative data analysis of over 1000 women has shown the mean sensitivity of stress echocardiography to detect physiologically significant coronary disease to be 81 %, with a specificity of 86 % [22, 27, 32]. Sensitivity range 75 %–93 % and specificity range 79 %–92 % to reliably detect multi-vessel disease with dobutamine stress echocardiography in women who are unable to exercise [22].

Stress echocardiography has been shown to be useful for determining cardiovascular prognosis in women [27–29]. In a study evaluating 5-year survival in 4234 women undergoing exercise stress echocardiography, survival in patients with no evidence of ischemia was found to be as high as 99.4 % [29]. Survival was found to be 97.6 % in women with evidence of single vessel ischemia and 95 % in women with evidence of multiple-vessel ischemia [29]. Although an abnormal stress echocardiogram correlates with a high cardiac event rate in women, a recent meta-analysis comparing dobutamine stress echocardiography to exercise single photon emission computed tomography (SPECT) myocardial perfusion imaging (MPI) revealed a yearly rate of 0.75 % of cardiovascular death and MI in the setting of a low-risk dobutamine echocardiogram, compared with 0.3 % per year in low-risk stress MPI [33]. The authors concluded that stress echocardiography may underestimate risk in women with less advanced CAD because perfusion abnormalities detected by SPECT MPI precede the wall-motion abnormalities detected by stress echocardiography. Wall motion abnormalities often occur in the setting of advanced stenosis and less predictably in myocardium supplied by mild-to-moderate coronary artery stenoses. Therefore, because an MI often presents in myocardial areas perfused by a less critical stenosis, stress echocardiography may underestimate cardiovascular risk in women with nonobstructive CAD [33].

The added imaging information provided by stress-echocardiography provides improved diagnostic and prognostic accuracy in women at risk for CAD compared with clinical variables and data from exercise treadmill testing [32]. In addition, a unique benefit of stress echocardiography for evaluating at-risk women is the absence of radiation exposure compared with other noninvasive imaging techniques (eg, SPECT, cardiac CT, coronary artery calcium scoring). Incremental data (ie, valvular function pericardial abnormalities) provided from a complete echocardiogram may also reveal an alternative explanation for symptoms of dyspnea or chest pain.

### Myocardial Perfusion Imaging with SPECT

Myocardial Perfusion Imaging with Single Photon Emission Tomography with ECG gating (MPI) provides quantitative information on myocardial perfusion, regional and global left ventricular function, and end-systolic and end diastolic volumes. The diagnostic and prognostic value of contemporary MPI techniques in evaluation of symptomatic women at risk for CAD has been supported by data [22, 34, 35]. The commonly seen limitations of traditional MPI techniques, including photon attenuation due to breast attenuation artifact and limited spatial resolution where minor perfusion defects may go undetected in smaller hearts [35, 36], are less problematic with the addition of electrocardiographic gating, attenuation correction protocols, use of prone imaging, and use of higher-energy radioisotope technetium [22, 37–39]. For the diagnosis of physiologically significant CAD in symptomatic women, the sensitivity of exercise contemporary MPI techniques ranges from 78 % to 88 %, with a specificity of 64 % to 91 % [37, 40].

An important advantage of MPI is the ability to use pharmacologic stress for at- risk patients who are unable to exercise or achieve an acceptable maximum heart rate with exercise. Pharmacologic vasodilator MPI has been shown to be accurate in detecting physiologically significant CAD in women with a sensitivity and specificity for coronary stenosis >50 % of 91 % and 86 %, respectively [41]. The importance of pharmacologic stress is relevant in daily clinical practice as very often, at-risk women are generally older and often have reduced exercise capacity when they present for evaluation. A large body of evidence supports the excellent prognostic accuracy of exercise and pharmacologic stress MPI in both men and women with the size and severity of defects directly linked to the risk of cardiac even independent of gender [35, 36, 42, 43].

The addition of gated left ventricular ejection fraction and left ventricular volume to perfusion data further enhances risk stratification [37, 38, 44, 45]. Pooled data in over 7000 women demonstrate an annual cardiac event rate of 0.7 % with a normal perfusion study [46]. Based on the available evidence, stress MPI is recommended for symptomatic women with an intermediate to high pretest likelihood of CAD with an abnormal, equivocal, or nondiagnostic baseline ECG. Pharmacologic stress is recommended for symptomatic women with normal or abnormal baseline ECG who are unable to exercise or unable to achieve maximum predicted target heart rate with exercise [45]. Stress MPI with exercise or pharmacologic stress adds incremental data to clinical variables and stress ECG data in symptomatic women at risk for IHD [43].

## Diagnostic Testing for Coronary Atherosclerosis: Beyond Epicardial Disease

For the symptomatic woman with a positive stress test and evidence of nonobstructive disease on coronary angiography, additional diagnostic testing may be warranted. Insights from clinical trials in the past decade have provided evidence to support the fact that several mechanisms contribute to worse outcomes and continued symptoms in the cohort of women who have no evidence of obstructive CAD.

Microvascular dysfunction of the coronary bed may in part account for the paradoxical finding of a greater symptom burden and high cardiac mortality in these women with a lower burden of obstructive CAD [14••]. Endothelial and smooth muscle cell dysfunction contribute to a microvascular disorder frequently observed in women with signs and symptoms of IHD and angiographically normal coronary arteries. Several theories have been proposed to explain normal angiographic results in women with chest pain. They include chest pain of noncardiac origin, coronary artery spasm, and diffuse disease without focal obstruction [47]. Higher rates of hypertension, left ventricular hypertrophy, and diabetes in women are hypothesized to result in a greater degree of microvascular disease in women compared with men with similar degrees of epicardial stenosis [14••, 15]. In the coronary tree, both endothelial-independent (microvascular) dysfunction and endothelial-dependent epicardial dysfunction have been shown to predict worse IHD events in patients undergoing diagnostic cardiac angiography, single-vessel percutaneous coronary angioplasty (PCI), or post ACS/MI [14••]. The identification of abnormalities in the coronary tree has implications in treatment strategies as restoration of endothelial function is associated with improved outcome. Improved endothelial function was associated with a 7.3-fold lower rate of cardiac events in a cohort of 400 hypertensive postmenopausal women, compared with women with no improvement [48]. Therefore, tests of vascular and endothelial integrity may help to demonstrate vascular dysfunction and identify women at high risk for cardiovascular outcomes [14••, 47]. Emerging data from contemporary imaging techniques with protocols and methods for the detection of subclinical atherosclerosis and endothelial dysfunction promise to reveal a new model for the identification and risk stratification of IHD in women [14••].

As we shift our focus from the detection of flow-limiting CAD lesions, cardiac imaging techniques with cardiac CT, positron emission tomography (PET), and CMR are emerging as techniques to investigate other contributors implicated in the spectrum of IHD in women—the coronary and noncoronary atherosclerotic burden. Also, other methods are being used with increasing frequency to detect dysfunction of the coronary endothelium and microvasculature.

### Coronary Artery Calcium Scoring and Computed Tomographic Angiography

Multi-slice computed tomography (CT) scanning with coronary artery calcium (CAC) scoring and computed tomographic angiography (CTA) allow for a noninvasive anatomic identification and quantification of obstructive and nonobstructive CAD. CAC detection adds incremental prognostic value to traditional cardiac risk factors for coronary artery disease [43, 49]. In an asymptomatic cohort of 4191 women and 6186 men, CAC was associated with a higher incidence of death in women than in men at each level of calcification: 80 % of women with extensive coronary calcification and CAC scores of more than 1000 were alive at 5-year follow-up compared with 98.4 % of women with no evidence of CAC [45]. CAC scoring is also highly sensitive for the presence of obstructive CAD (≥50 % stenosis) and provides an estimate of the total calcified atherosclerotic plaque burden, thereby correlating to a patient’s cardiac risk [50]. Sex-specific analysis of the role of CAC in evaluating symptomatic women at risk for coronary disease reveals a high negative predictive value when correlated with invasive coronary angiography. Of 539 symptomatic women who underwent clinically indicated coronary angiography, 41 % had a normal angiogram without evidence of CAC, demonstrating a negative predictive value of 100 %. There was a greater prevalence of obstructive CAD in women with CAC scores of more than 100 [45].

Recent data reveal an evolving role for cardiac CT in the identification and risk stratification of CAD in women at risk for coronary disease [45, 51]. CTA allows for a noninvasive evaluation of the coronary arteries with high diagnostic accuracy for obstructive epicardial CAD [52, 53]. In a series of 51 women and 52 men, the diagnostic sensitivity and specificity was similar by sex at 85 % and 99 %, respectively [54]. However, one of the important limitations of CTA, particularly in young women, is the potential increase in lifetime risk of cancer due to concentrated ionizing radiation to radiosensitive breast tissue [55]. Consequently, considerable efforts in dose reduction strategies are being employed in order to reduce radiation while optimizing diagnosis of atherosclerosis in symptomatic patients at risk for CAD [56, 57].

### Myocardial Perfusion Imaging with Positron Emission (PET)

MPI with PET is a robust nuclear medicine imaging technique, and with its superior spatial resolution improves on the diagnostic and prognostic accuracy for detecting epicardial CAD in both men and women. Given the spectrum of IHD in women, an enhanced value to PET is the ability to calculate absolute blood flow in all areas of the coronary tree, assess wall motion at peak hyperemia with vasodilator stress, and evaluate coronary flow reserve, thereby interrogating the coronary microvasculature and endothelium [58–60]. The capacity to quantify myocardial perfusion provides an added advantage over SPECT for evaluating multi-vessel CAD [60, 61]. Multiple studies have performed direct comparison of the diagnostic accuracy of 82Rb myocardial perfusion PET and 201Tl or 99mTc SPECT. Their overall results showed a high sensitivity of 90 %, high specificity of 86 % and diagnostic accuracy of 90 % with PET [60, 61]. Vasodilatory stress MPI with PET using nuclear tracer 82Rb allows for integrated photon attenuation correction and enhanced image quality, a notable advantage over SPECT, when evaluating CAD in obese women. The use of the isotope 82Rb PET is additionally beneficial in women due to the ability to accurately quantify absolute values of regional and global myocardial blood flow to assess microvascular disease (flow reserve) [39]. Moreover, contemporary integrated hybrid PET/CT offers an opportunity to assess the presence and magnitude of subclinical atherosclerotic disease burden and to further investigate myocardial blood flow as a marker of endothelial function and atherosclerotic disease activity which is of paramount importance in female patients [60].

As with SPECT, increasing extent and severity of perfusion defects with stress PET is associated with increasing frequency of adverse events. The overall event rate in patients with normal stress PET was 0.4 % per year [60]. A recent study on a cohort of 1432 patients followed for 1 year after 82Rb PET MPI with vasodilatory stress was shown to be a powerful predictor of cardiac event and survival in patient with known CAD or an intermediate to high pretest likelihood of CAD [58]. Myocardial perfusion imaging added incremental value to LVEF (ie, at any LVEF, a higher summed stress score had greater risk) and LVEF added incremental value to myocardial perfusion imaging (ie, at any summed stress score, a lower LVEF had greater risk). Furthermore, LVEF reserve independently provided significant and incremental value over perfusion for predicting future risk of adverse events [58, 60].

With its ability to detect epicardial disease as well as evaluate the coronary microvasculature and endothelium, this technology is an important advance in imaging, specifically for imaging of CAD in women. Future investigations of the potential complementary role of CTA and PET perfusion imaging for risk stratification and management of women with IHD promise to expand PET’s role to include an evaluation of the atherosclerotic burden.

### Cardiac Magnetic Resonance Imaging (CMR)

Cardiac magnetic resonance imaging (CMR) is emerging as an important imaging modality for the diagnosis of CAD in women. CMR allows for the differentiation of a range of myocardial diseases ranging from ischemic heart disease to nonischemic cardiomyopathies, including stress-induced cardiomyopathy (takotsubo) [62–65]. CMR perfusion has the unique ability to evaluate subendocardial ischemia, assess left and right ventricular function, and provide a detailed anatomic evaluation of both the myocardium as well as of the peripheral vasculature. The specific advantages to the use of CMR imaging for the evaluation of women with suspected CAD is its excellent soft tissue characterization, three-dimensionality, superior temporal and spatial resolution, and lack of ionizing radiation [4, 66].

Dobutamine CMR has been studied to identify flow limiting coronary artery stenoses in women [66]. In a study of a cohort of 266 women, inducible left ventricular wall motion abnormalities during dobutamine CMR predicted cardiac death and MI in women with known or suspected ischemic heart disease [67]. Although data sets are small, the results suggest that dobutamine CMR is a valuable noninvasive stress imaging modality for identifying disease in at-risk women, with a sensitivity and specificity for detecting obstructive CAD in women of 85 % and 86 %, respectively [68].

CMR with vasodilator stress (dipyridamole or adenosine) has also been used to detect myocardial ischemia through first-pass perfusion imaging [69–73]. Ischemic territories can be identified through intravenous dipyridamole and gadolinium for first-pass perfusion and an estimation of a myocardial perfusion reserve index. In a small study of 48 patients and 18 healthy subjects, a coronary flow reserve less than 1.65 had a sensitivity and specificity of 91 % and 94 % percent, respectively, for the detection of coronary artery disease as defined by PET scanning [72].

There is growing evidence on the prognostic ability of CMR in symptomatic women at risk for IHD. In one of the first reports assessing the prognostic value of CMR with adenosine and dobutamine, 513 patients were evaluated during a mean follow-up of 2.3 years [74]. The 3-year event-free (cardiac death and nonfatal myocardial infarction) survival was 99 % with normal and 84 % with abnormal combined stress images. CMR has added value for symptomatic women who are found to have nonobstructive or normal coronaries on invasive angiography. Due to its excellent temporal and spatial resolution, CMR with perfusion has the ability to identify subendocardial ischemia and may provide an etiology for women with persistent chest pain without obstructive CAD [66]. In addition, CMR has the ability to detect prior or recent myocardial infarctions with high sensitivity, which may be silent or unsuspected in women who are at risk.

## Additional Emerging Imaging Techniques for Detection of IHD in Women

Emerging innovative techniques have the ability to provide semiquantitative information of coronary flow, with slow flow in the absence of significant stenosis reflect impairment of the myocardial microcirculatory perfusion [75]. The use of intravascular coronary ultrasound and intracoronary Doppler flow wire has essentially replaced the use of acetylcholine infusion for the evaluation of endothelial function. Coronary flow can be studied invasively in the cardiac catheterization laboratory by using intracoronary Doppler wires or by the thrombolysis in myocardial Infarction frame count to measure flow [76]. For the woman with ischemic symptoms, an abnormal stress test with no evidence of flow-limiting stenoses on standard angiography, intravascular ultrasonography, or intracoronary Doppler flow may document atherosclerosis within the arterial wall. This is relevant as significant “outward remodeling” remodeling, atherosclerotic lesions that protrude outward rather than impinging on the lumen as seen in classic obstructive disease) with impaired coronary flow reserve or endothelial dysfunction has been noted to occur more frequently in women and be the cause of symptoms [11].

Emerging noninvasive methods for the detection of endothelial dysfunction in addition to those discussed above include high-frequency transthoracic Doppler harmonic echocardiography, transesophageal echocardiography with Doppler imaging, and digital reactive hyperemia peripheral arterial tonometry [77]. Brachial artery reactivity testing offers a window to study the integrity of the coronary endothelium and can be used to noninvasively detect endothelial dysfunction as impaired brachial endothelial function is associated with increased cardiovascular adverse events.

Recent evidence point to the presence of atherosclerosis in the peripheral vasculature as highly correlated with coronary atherosclerosis. The use of ABI and CIMT are noninvasive imaging modalities that can be used for detection of peripheral atherosclerosis as a surrogate for CAD in asymptomatic women at risk for IHD [14••].

## Recommendations

It is important for clinicians to appreciate that although women have a higher atherosclerotic burden, are more symptomatic, and have a worse clinical outcome, they have a lower prevalence of obstructive coronary disease than men. The pathophysiology of heart disease in women is a spectrum and, therefore, the clinician must consider a unique evaluation approach, which in some case will extend beyond the detection of epicardial stenosis to include evaluation of the atherosclerotic burden as well as an evaluation of coronary reactivity of the microvasculature and endothelium.

Clinicians must evaluate risk factors, symptoms, and baseline ECG in women with risk factors for IHD to establish their pretest likelihood of disease. Next, an assessment of the patient’s functional capacity is important to assess prognosis, but also to appropriately choose the best noninvasive stress testing modality. Current evidence supports the use of the exercise ECG stress test as the initial test for symptomatic women with a normal resting ECG and good exercise tolerance (capable of >5 METS). The calculation of functional capacity and clinical scores such as the Duke Treadmill Score further improve the ability to diagnosis and to assess prognosis in women [4, 22].

**Fig. 1:**
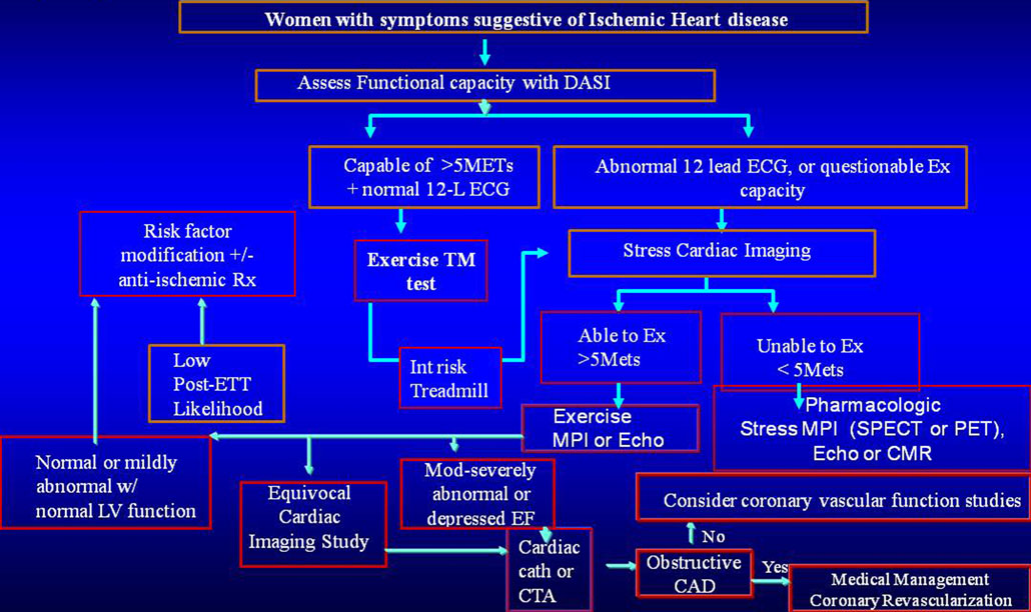
Adapted with permission from Phillips L.M. Noninvasive Assessment of Coronary Artery Disease in Women: What’s Next?. Curr Cardio Reports. 2010;12:152

Cardiac imaging with stress MPI or stress echocardiography provides incremental information over clinical variables and the exercise ECG in symptomatic women with suspected CAD. Local expertise should guide selection of the test. According to the American Heart Association consensus statement, symptomatic women with questionable exercise capacity, abnormal baseline ECG, and those with diabetes mellitus, should undergo cardiac imaging with exercise or pharmacologic stress as the initial test in the evaluation of symptoms [24•]. Evolving evidence supports the use of newer techniques like CTA in the setting of an abnormal or equivocal stress cardiac imaging study. PET and CMR are other newer methods that can be very useful in symptomatic women with no evidence of obstructive CAD to evaluate the coronary microvasculature for evidence of subendocardial ischemia or abnormal coronary reserve (Fig. 1).

## Conclusions

Insights from recent clinical trials have provided evidence that there are clear gender differences that exist in the pathophysiology, clinical presentation, diagnosis, and treatment of CAD. The etiology of IHD in women is multifactorial and includes a spectrum from obstructive epicardial CAD to nonobstructive CAD with dysfunction of the coronary microvasculature and endothelium. Future imaging protocols that focus on analyzing endothelial function and detection of subclinical atherosclerosis will likely be integrated into diagnostic and prognostic algorithms for at-risk women. Emerging novel technologies and protocols using CMR, assessment of carotid intima-media thickness, and brachial artery flow-mediated dilatation will be useful to further identify atherosclerotic burden and define the unique pathophysiology of ischemic heart disease in symptomatic women without evidence of obstructive CAD.
